# Full Implementation of the Secondary 1 Program of Project P.A.T.H.S.: Observations Based on the Co-Walker Scheme

**DOI:** 10.1100/tsw.2009.116

**Published:** 2009-10-01

**Authors:** Daniel T. L. Shek Shek, Rachel C. F. Sun, Vivian W. M. Kan

**Affiliations:** ^1^ Department of Applied Social Sciences, Hong Kong Polytechnic University, Hong Kong, P.R.C., China; ^2^ Department of Sociology, East China Normal University, Shanghai, P.R.C., China; ^3^ Kiang Wu Nursing College of Macau, Macau, P.R.C., China; ^4^ Department of Social Work, Chinese University of Hong Kong, Hong Kong, P.R.C., China

**Keywords:** quality of curriculum delivery, observation, positive youth development program

## Abstract

This study was conducted in order to understand the implementation quality of the Secondary 1 Program of the Tier 1 Program of the Project P.A.T.H.S. (Positive Adolescent Training through Holistic Social Programmes) in the first year of the Full Implementation Phase. Classroom observations of 137 units in 85 schools were conducted under the Co-Walker Scheme. Results showed that the overall level of program adherence was generally high, with an average of 86.57%. Thirteen aspects concerning program delivery were significantly correlated. Multiple regression analyses revealed that (1) overall implementation quality was significantly predicted by interactive delivery method, use of positive and supportive feedback, opportunity for reflection, degree of achievement of the objectives, and lesson preparation; whereas (2) success of implementation was significantly predicted by student interest, interactive delivery method, use of positive and supportive feedback, opportunity for reflection, and degree of achievement of the objectives. In general, the present study suggests that the implementation quality of the Project P.A.T.H.S. is good.

## INTRODUCTION

To promote holistic development among adolescents in Hong Kong, The Hong Kong Jockey Club Charities Trust approved HK$400 million to launch a project entitled “P.A.T.H.S. to Adulthood: A Jockey Club Youth Enhancement Scheme”. Project P.A.T.H.S. (**P**ositive **A**dolescent **T**raining through **H**olistic **S**ocial Programmes) is a two-tier program, of which the Tier 1 Program is a universal positive youth development program involving the participation of Secondary 1 to 3 students, while the Tier 2 Program is a selective program designed for students with greater psychosocial needs.

There are three main components in the Project P.A.T.H.S.: curriculum development, training, and evaluation. First, the research team developed a set of program manuals based on 15 positive youth development constructs proposed by Catalano and colleagues[1], as well as adolescent development needs, Chinese cultural values, and local youth issues[2,3]. The manuals are used by school teachers and social workers to implement the 20-h Tier 1 Program to students at each grade. Second, a 3-day training workshop is provided to school teachers and social workers to familiarize them with the program design and philosophy, and to provide them with an opportunity to reflect on their beliefs about positive youth development prior to program implementation[4]. Third, several evaluation strategies were adopted to assess program effectiveness, including qualitative evaluation, such as focus groups[5,6]; students' weekly diary analysis[7] and case studies[8,9]; longitudinal objective outcome evaluation[10,11]; subjective outcome evaluation assessing students' and instructors' perceptions of the program[12,13,14,15,16]; as well as process evaluation, including observation[17,18] and interim evaluation[19,20]. All these findings consistently demonstrated that the Tier 1 Program could enhance students' psychosocial development, and was well received by the instructors and students.

In addition, the research team of the Project P.A.T.H.S. introduced a Co-Walker Scheme in the 2006/07 school year in order to cultivate a supportive atmosphere between front-line workers and the research team, without any monitoring and evaluating purposes. Each participating school is assigned a co-walker, who is a colleague of the project, acting as the resource person and communication bridge. The co-walkers visit schools, observe program delivery in the classroom, and give necessary support and advice to instructors, in order to ensure that program delivery in each school is acceptable. At the same time, the instructors are encouraged to share their experiences on program implementation, and make suggestions and recommendations for modifying the program. In short, a supportive partnership is built between the program implementers and the research team during program implementation.

To evaluate such a well-designed program, the importance of conducting process evaluation to move beyond the investigation on "does the program work?" to "how does the program work?" is advocated[21,22,23,24,25]. According to Scheirer[26], "process evaluation is the use of empirical data to assess the delivery of programs…. Process evaluation verifies what the program is, and whether or not it is delivered as intended to the targeted recipients and in the intended dosage" (p. 40). Moreover, process evaluation allows investigation of the context where the program is being carried out, adequacy of program content and design, and quality of delivery[21,22,23,24,25]. All of these not only help to explain program outcomes, but also help to identify factors leading to successful program implementation, and thus suggesting ways to improve and sustain the program. Therefore, it is worthwhile to conduct process evaluation in order to examine the significant process variables relating to program success.

Among various potential process variables that influence program implementation, program adherence and fidelity are the key variables of quality implementation, according to Fagan and Mihalic[27]. Moreover, high student interest and involvement, as well as well-trained instructors with good quality, were found to be the key ingredients of an effective program[28,29,30]. In addition, instructors who are familiar with their students, accept their students, and believe in their potentials[31], and who are well prepared and familiar with the teaching materials[28], were found to contribute to favorable program outcomes. More importantly, using multiple instructional strategies to deliver the program (e.g., proactive classroom management, cooperative learning methods, strategies to enhance student motivation) was found to be a significant facilitating factor leading to program success[21]. In contrast, if instructors are unable to adopt participatory teaching methods (e.g., drama, role play, and group discussion) or do not have innovative instructional skills to implement the program in the classroom, it will adversely affect the program implementation quality[23,24]. All these findings highlight the necessity to examine program adherence, students' participation, instructors' quality, classroom management, and delivery skills as process variables relating to the overall program implementation quality and success.

To assess these program variables, systematic classroom observation is considered to act as a vital means[32,33]. In Project P.A.T.H.S., process evaluation based on systematic classroom observation was carried out that showed that program adherence was high (about 80–85%) and the program was generally implemented successfully in the observed schools[17,18]. However, the available process evaluation studies in the project were based on a relatively small proportion of schools (about 6–8% of the participating schools) randomly selected from the participating schools. Because about 41% of the participating schools that implemented the Secondary 1 Program were observed by co-walkers in the first year of the Full Implementation Phase in the 2006/07 school year, evaluation based on such a larger sample size allows higher generalizability of the findings. Moreover, the data collected in the Co-Walker Scheme provide a detailed description of selected features of activities and interactions in classroom, although they are mainly used for administrative purpose. To utilize these data fully, the present study attempted to examine (1) the quality of program implementation, particularly program adherence in accordance with the program manuals; (2) the relationships among different aspects of program delivery; and (3) the predictors of overall implementation quality and implementation success. Based on literature review and the findings obtained in the Secondary 2[34] and Secondary 3 Program[35], it was hypothesized that higher student interest and participation, better lesson preparation, better classroom and time management, and better program delivery strategies would be related to higher implementation quality and success.

## METHODS

### Participants

Among the 207 schools that joined the Secondary 1 Program of the Project P.A.T.H.S. in the first year of the Full Implementation Phase in the 2006/07 school year, there were 112 schools that adopted the full program (i.e., 20-h program involving 40 units) and 95 schools that adopted the core program (i.e., 10-h program involving 20 units). Among these schools, 85 schools (55 that adopted the full program and 30 that adopted the core program) were observed under the Co-Walker Scheme (Table 1). There were 112 schools not observed due to school rejection and time limitation. In short, about 41% of the participating schools were observed in the present study.

**TABLE 1**
**Basic Information of Observed Schools**

| Basic Information | Hours of Training | | |
|---|---|---|---|
| | 10 | 20 | Total |
| Total number of schools observed | 30 | 55 | 85 |
| Total number of units observed | 44 | 93 | 137 |
| Number of units observed per school | 1–2 | 1–4 | 1–4 |
| Average number of students per class | 37.14 | 36.50 | 36.71 |
| Average number of instructors per class | 2.3 | 2.08 | 2.15 |
| Average duration of observation (min) | 37.16 | 37.53 | 37.41 |

*Note:* The observed units of the Secondary 1 Program covered 14 positive youth development constructs, i.e., bonding, social competence, emotional competence, cognitive competence, behavioral competence, moral competence, self-efficacy, prosocial norms, resilience, self-determination, spirituality, clear and positive identity, beliefs in the future, and prosocial involvement.

## Instrument

A rating form was designed for each observer to record how each teaching unit was implemented in the classroom. It includes four major areas: basic information of the class, integration with the school formal curriculum, program fidelity and adherence, and quality of program delivery. For program fidelity and adherence, the observers were required to rate the degree of adherence and record the time used to implement the unit. The Curriculum Delivery Assessment Scale was used to measure the quality of program delivery in the areas of student interest, student participation and involvement, classroom control, use of interactive delivery method, use of strategies to enhance student motivation, use of positive and supportive feedback, instructors' familiarity with the students, opportunity for reflection, degree of achievement of the objectives, time management, quality of preparation, overall implementation quality, and success of implementation. The rating form also includes three open-ended questions for the observers to fill in further information. These included their feelings towards the lesson, other feelings or observations, and comments made by the instructors.

## Procedures

School consent was obtained prior to the study, which was carried out from December 2006 to May 2007. Each teaching unit was observed by one observer. The observers were six colleagues of the Project, including five registered social workers and one postdoctoral fellow. Before conducting the observational study, the observers were trained to standardize the data collection procedure and rating of classroom observation, so as to ensure the quality and consistency of the data collected.

## RESULTS

As shown in Table 1, systematic observation of one to two teaching units in schools that adopted the core program and one to four teaching units in schools that adopted the full program was conducted. There were 137 units under observation, which covered 14 positive youth development constructs: bonding, social competence, emotional competence, cognitive competence, behavioral competence, moral competence, self-efficacy, prosocial norms, resilience, self-determination, spirituality, clear and positive identity, beliefs in the future, and prosocial involvement. The average duration of observation was 37.41 min/unit. The average number of students and instructors per class were 36.71 and 2.15, respectively.

As shown in Table 2, a majority of the observed units was incorporated into the school's formal curriculum (70.8%), such as Life Education, Civic Education, Liberal Studies, Integrated Humanities, Moral Education, Social Studies, Personal Growth, and Religious Studies. About one-fourth of observed units were implemented outside the formal curriculum (24.1%), such as after school hours and during holidays, teachers' periods, postexamination days, assemblies, or camps.

Reliability analysis showed that the Curriculum Delivery Assessment Scale was highly reliable ($\alpha$ = 0.96; mean interitem correlations = 0.66; Table 3). To obtain an overall picture, the ratings for each item across all units were averaged. Results in Table 3 revealed that the mean rating of overall implementation quality was high (5.60 on a 7-point rating scale) and the implementation of the program was also considered to be successful (5.66 on a 7-point rating scale). An examination of different curriculum delivery aspects showed that the mean ratings were generally high (over 5 on a 7-point rating scale), particularly on lesson preparation (over 6 on a 7-point rating scale).

In addition, the average overall adherence to the curriculum manuals was 86.57% (range: 20–100%), which was quite remarkable (Table 3). For the observed units, where modifications had been made, the observers generally regarded the changes to be reasonable. However, the degree of adherence of an observed unit was rated to be very low (i.e., 20%). An examination of the observer's written comments

**TABLE 2**
**The Percentage of Observed Tier 1 Program Units Implemented in Different Modes for Schools Adopting 10 and 20 Hours of Implementation**

| Different Mode | Hours of Implementation | | |
|---|---|---|---|
| | 10 | 20 | Total |
| Incorporated into the formal curriculum | 35 (79.5%) | 62 (66.7%) | 97 (70.8%) |
| Outside formal curriculum | 9 (20.5%) | 24 (25.8%) | 33 (24.1%) |
| Incorporated into formal curriculum and outside formal curriculum | 0 (0%) | 6 (6.5%) | 6 (4.4%) |
| Incorporated into formal curriculum and other time slots | 0 (0%) | 1 (1.1%) | 1 (0.7%) |
| Total | 44 (100%) | 93 (100%) | 137 (100%) |

*Note:* (1) Formal curriculum included Life Education, Civic Education, Liberal Studies, Integrated Humanities, Moral Education, Social Studies, Personal Growth, and Religious Studies. (2) Outside formal curriculum refers to the implementation after school, during holidays, teachers' periods, postexam days, assemblies, or camps.

**TABLE 3**
**Cronbach Alpha Coefficients, Means and Standard Deviations of the Curriculum Delivery Assessment Scale, and Average Adherence Rate**

| Quality of Curriculum Delivery | Corrected Item-Total Correlation | Total Alpha if Item is Deleted | Mean | Standard Deviation |
|---|---|---|---|---|
| 1. Student interest | 0.84 | 0.96 | 5.52 | 1.13 |
| 2. Student participation and involvement | 0.84 | 0.96 | 5.61 | 1.15 |
| 3. Classroom control | 0.81 | 0.96 | 5.69 | 1.19 |
| 4. Interactive delivery method | 0.84 | 0.96 | 5.50 | 1.11 |
| 5. Strategies to enhance student motivation | 0.76 | 0.96 | 5.40 | 1.09 |
| 6. Use of positive and supportive feedback | 0.85 | 0.96 | 5.54 | 1.09 |
| 7. Instructors' familiarity with the students | 0.59 | 0.96 | 5.55 | 1.34 |
| 8. Opportunity for reflection | 0.79 | 0.96 | 5.12 | 1.08 |
| 9. Degree of achievement of the objectives | 0.85 | 0.96 | 5.67 | 0.98 |
| 10. Time management | 0.71 | 0.96 | 5.70 | 0.97 |
| 11. Lesson preparation | 0.64 | 0.96 | 6.03 | 1.00 |
| 12. Overall implementation quality | 0.92 | 0.96 | 5.60 | 1.12 |
| 13. Success of implementation | 0.92 | 0.96 | 5.66 | 1.08 |
| | Cronbach's Alpha = 0.96 | | Average Adherence = 86.57% | |

showed that there was great modification on the program design, teaching materials, and instructional format in that lesson, which deviated from the original lesson plan.

> *"Although the teacher prepared a lot of audio-visual teaching materials, there was too much information that made it hard for the students to grasp the main points. The teacher made great modifications and thus the original design and learning targets were lost. The lesson duration was long, but there was no small group discussion for students. Students'*

*involvement was very low, and the teaching was one-way. The lesson was overloaded with lots of information."*

In Table 4, results of Pearson correlation analyses showed that all 13 items (student interest, student participation and involvement, classroom control, interactive delivery method, strategies to enhance student motivation, use of positive and supportive feedback, instructors' familiarity with the students, opportunity for reflection, degree of achievement of the objectives, time management, lesson preparation, overall implementation quality, and perceived successfulness of program implementation) were positively correlated. Particularly, the overall implementation quality (item 12) and success of implementation (item 13) were highly correlated (r = 0.94, $p < 0.01$). Moreover, both were significantly and positively correlated with all the other items, and had relatively high correlations with interactive delivery method (item 4, r = 0.81 and r = 0.82, $p < 0.01$), use of positive and supportive feedback (item 6, r = 0.80 and r = 0.81, $p < 0.01$), and degree of achievement of the objectives (item 9, r = 0.84 and r = 0.82, $p < 0.01$).

**TABLE 4**
**Intercorrelations among Items of the Curriculum Delivery Assessment**

| Item | 1 | 2 | 3 | 4 | 5 | 6 | 7 | 8 | 9 | 10 | 11 | 12 | 13 |
|---|---|---|---|---|---|---|---|---|---|---|---|---|---|
| 1. Student interest | 1.00 | | | | | | | | | | | | |
| 2. Student participation and involvement | 0.85** | 1.00 | | | | | | | | | | | |
| 3. Classroom control | 0.79** | 0.77** | 1.00 | | | | | | | | | | |
| 4. Interactive delivery method | 0.71** | 0.78** | 0.67** | 1.00 | | | | | | | | | |
| 5. Strategies to enhance student motivation | 0.69** | 0.71** | 0.63** | 0.72** | 1.00 | | | | | | | | |
| 6. Use of positive and supportive feedback | 0.72** | 0.71** | 0.78* | 0.69** | 0.71** | 1.00 | | | | | | | |
| 7. Instructors' familiarity with the students | 0.49** | 0.45** | 0.45** | 0.58** | 0.46** | 0.61** | 1.00 | | | | | | |
| 8. Opportunity for reflection | 0.67** | 0.71** | 0.68** | 0.73** | 0.63** | 0.64** | 0.50** | 1.00 | | | | | |
| 9. Degree of achievement of the objectives | 0.72** | 0.68** | 0.68** | 0.72** | 0.60** | 0.72** | 0.57** | 0.72** | 1.00 | | | | |
| 10. Time management | 0.60** | 0.55** | 0.58** | 0.56** | 0.55** | 0.64** | 0.46** | 0.64** | 0.72** | 1.00 | | | |
| 11. Lesson preparation | 0.51** | 0.50** | 0.49** | 0.55** | 0.48** | 0.59** | 0.39** | 0.47** | 0.66** | 0.55** | 1.00 | | |
| 12. Overall implementation quality | 0.78** | 0.79** | 0.76** | 0.81** | 0.70** | 0.80** | 0.57** | 0.77** | 0.84** | 0.64** | 0.67** | 1.00 | |
| 13. Success of implementation | 0.80** | 0.80** | 0.79** | 0.82** | 0.71** | 0.81** | 0.56** | 0.79** | 0.82** | 0.66** | 0.59** | 0.94** | 1.00 |

*Note:* **$p < 0.05$, *$p < 0.01$.

Based on these findings, separate multiple regression analyses were performed in order to examine the contribution of the 11 aspects of program delivery to (1) overall implementation quality and (2) success of implementation. Results in Table 5 show that the overall implementation quality was significantly predicted by interactive delivery method ($\beta = 0.16$, $p < 0.05$), use of positive and supportive feedback ($\beta = 0.19$, $p < 0.01$), opportunity for reflection ($\beta = 0.16$, $p < 0.01$), degree of achievement of the objectives ($\beta = 0.26$, $p < 0.01$), and lesson preparation ($\beta = 0.14$, $p < 0.01$). The model explained for 86% of the variance in overall implementation quality [$F(11, 134) = 69.41$, $p < 0.01$]. Similarly, success of implementation was significantly predicted by student interest ($\beta = 0.16$, $p < 0.05$), interactive delivery method ($\beta = 0.22$, $p < 0.01$), use of positive and supportive feedback ($\beta = 0.22$, $p < 0.01$), opportunity for reflection ($\beta = 0.17$, $p < 0.01$), and degree of achievement of the objectives ($\beta = 0.21$, $p < 0.01$). The model explained for 86% of the variance in implementation success [$F(11, 134) = 69.77$, $p < 0.01$].

**TABLE 5**
**Summary of the Multiple Regression Analyses**

| Overall Implementation Quality | | Success of Implementation | |
|---|---|---|---|
| **Significant Predictors** | **Standardized β** | **Significant Predictors** | **Standardized β** |
| 1. Interactive delivery method | 0.16* | 1. Student interest | 0.16* |
| 2. Use of positive and supportive feedback | 0.19** | 2. Interactive delivery method | 0.22** |
| 3. Opportunity for reflection | 0.16** | 3. Use of positive and supportive feedback | 0.22** |
| 4. Degree of achievement of the objectives | 0.26** | 4. Opportunity for reflection | 0.17** |
| 5. Lesson preparation | 0.14** | 5. Degree of achievement of the objectives | 0.21** |
| | $R^2 = 0.86$ | | $R^2 = 0.86$ |
| | $F(11, 134) = 69.41$** | | $F(11, 134) = 69.77$** |

*Note*: $^{**}p < 0.01$, $^{*}p < 0.05$.

## DISCUSSION

The purposes of the present study were threefold: (1) to examine the quality of program implementation, particularly program adherence in accordance with the program manuals; (2) to investigate the relationships among different aspects of program delivery; and (3) to explore the predictors of overall implementation quality and implementation success. Several prominent observations could be highlighted from the findings. First, the present study showed that the overall degree of adherence to the program manuals assessed by the co-walkers was high (86.6%). The result was generally consistent with that assessed by independent observers in the process evaluation (86.3%)[18] and that reported by instructors in subjective outcome evaluation (87.1%)[36]. In short, the findings once again support the view that the need for modifying the teaching units of the Tier 1 Program was not high. Because the program was systematically designed according to theoretical concepts and research findings[1], and addressed various adolescent developmental needs and issues[2,3], drastic modification would adversely affect the intended learning outcomes. Moreover, as adequate teaching materials were designed for each unit, which had been trial-taught in schools to assure the quality and practicability of the program across different school contexts and adolescent populations, the present findings reinforce the argument that the program can be easily adopted without much adaptation.

Second, as school policy governing program integration into the formal curriculum was found to be a vital process variable facilitating program implementation[8], the present findings suggest that it is feasible to incorporate this positive youth development program into school formal subjects, such as Life Education, Civic Education, Liberal Studies, Integrated Humanities, Moral Education, Social Studies, and Religious Studies, which are related to students' psychosocial and spiritual development. By integrating positive youth development programs into the school formal curriculum, the status of implementing youth programs in schools can be solidified, and both the instructors' motivation in program delivery and students' participation can be raised.

Third, in line with the results obtained in process evaluation[17,18], different aspects of the program delivery were perceived to be very positive. These aspects include (1) students' interest and involvement (items 1 and 2); (2) instructors' effort, classroom management skills, and teaching strategies (items 3, 4, 5, 6, 8, 10, and 11); and (3) instructors' relationship with the students (item 7). Also, the co-walkers perceived that the objectives of the units implemented could be achieved (item 9), and the implementation was good and successful (items 12 and 13). Generally, the present findings indicate that the Secondary 1

students enjoyed the program, and the instructors could deliver the program as intended with adequate instructional strategies. Among all, lesson preparation (item 11) was rated the highest, which indicates that the instructors were responsible and well prepared for the program delivery. Since the Project P.A.T.H.S. was a pioneering positive youth development program adopted by the schools, instructors might need to pay double effort to become accustomed to this new program in the first year of the Full Implementation Phase. Although some instructors indicated that additional preparatory work increased their workload[19,20], they still regarded the implementation of such a positive youth development program as meaningful and beneficial to students[8]. Therefore, instructors' effort in program delivery should deserve appreciation and recognition.

Fourth, different aspects of program delivery were significantly correlated, as hypothesized. Multiple regression analyses revealed that both overall implementation quality and success of implementation were significantly predicted by interactive delivery method, use of positive and supportive feedback, opportunities for reflection, and the degree of achievement of the objectives. These findings revealed that "instructor" is the most critical "people" factor because how they deliver the program would greatly influence the implementation quality and program success. Particularly, instructors who are able to deliver the program in an interactive manner, and provide positive and supportive feedback, can engage students' participation and stimulate students' learning[37]. Moreover, as reflection is an indispensable component in experiential learning, instructors who can create rooms for students to reflect on what had been learned and how it could apply to daily life can enhance students' personal growth. All these are invaluable process variables contributing to better implementation quality and program success, and thus reaching the ultimate goal of enhancing adolescents' holistic development.

Furthermore, echoing the importance of guaranteeing program adherence, this study also showed that the program would be perceived as good and successful when the program objectives could be highly achieved. Although some modifications in delivery methods during program implementation are allowed to cater to students with different learning needs, it is necessary to ensure that the intended messages and skills were transmitted to the students. Otherwise, too much deviation from the original lesson plans and learning targets would spoil delivery quality and inhibit program success, as elaborated in the co-walker's written comments.

In addition, lesson preparation was found to predict the overall implementation quality significantly. Consistent with previous findings[34,35], instructors' effort in preparing the lesson well was found to increase the overall program implementation quality. It might be particularly true when the program is in the initial implementation phase, where instructors have to prepare lessons, as well as themselves, well enough to guarantee smooth program delivery.

Besides, student interest was found to contribute significantly to program success. Similar to previous findings[28,31], the present study demonstrated that "student" is another important "people" factor facilitating program success. Particularly, when students like to attend the program and enjoy the games, role playing, and group discussions, it helps to create a happy learning environment that enables implementation success.

In sum, the present process evaluation study identifies six important inter-related factors that may explain the perceived successfulness of the program with good implementation quality. The first factor refers to program adherence and achievement of program objectives. The other four factors relate to instructors' program delivery skills and preparation, i.e., to carry out the program in an interactive manner, to give positive feedback and support to students, to create space for students' reflection, and to prepare well for the lessons. The last factor is student interest in attending the lessons. By identifying these successful factors in program delivery, it sheds light on how further effort could be made to improve the program implementation quality, for instance, reminding instructors of the importance of program fidelity via the Co-Walker Scheme, and equipping instructors with the above-mentioned skills via instructor training workshops.

Although large sample size (41% of the participating schools) serves as the strength of the present study, there were several limitations that should not be overlooked. First, the sample was not randomly selected and thus the findings might not be truly representative of the schools participating in the project.

Second, it may be criticized that the observation findings might be rather subjective because each lesson was observed by one observer only. However, this criticism can be partially dismissed as the observers had been trained. Third, some variables affecting the implementation might be missed in the classroom observation. For instance, the personality of teachers may affect the implementation, but this variable cannot be observed in the classroom. Fourth, in this cross-sectional study, no causal relationship could be shown. Further observation could be conducted in each school across different time points so as to collect longitudinal data for testing the predictive contributions of program fidelity and curriculum delivery to program success. Despite the above limitations, the present findings are encouraging, and suggest that the Tier 1 Program of the Project P.A.T.H.S. (Secondary 1 Program) was implemented successfully in the first year of the Full Implementation Phase.

## ACKNOWLEDGMENTS

The preparation for this paper and the Project P.A.T.H.S. were financially supported by The Hong Kong Jockey Club Charities Trust.
